# Sequential Changes in the Mesenteric Lymph Node Microbiome and Immune Response during Cirrhosis Induction in Rats

**DOI:** 10.1128/mSystems.00278-18

**Published:** 2019-02-19

**Authors:** Alba Santiago, Elisabet Sanchez, Allison Clark, Marta Pozuelo, Miguel Calvo, Francisca Yañez, Guillaume Sarrabayrouse, Lidia Perea, Silvia Vidal, Alberto Gallardo, Carlos Guarner, German Soriano, Chaysavanh Manichanh

**Affiliations:** a Department of Gastroenterology, Vall d'Hebron Research Institute, Barcelona, Spain; b Department of Gastroenterology, Hospital de la Santa Creu i Sant Pau, Universitat Autònoma de Barcelona, Barcelona, Spain; c Department of Immunology, IIB-Sant Pau Research Institute, Hospital de la Santa Creu i Sant Pau, Universitat Autònoma de Barcelona, Barcelona, Spain; d CIBERehd, Instituto de Salud Carlos III, Madrid, Spain; e Department of Pathology, Hospital de la Santa Creu i Sant Pau, Universitat Autònoma de Barcelona, Barcelona, Spain; University of Colorado Denver

**Keywords:** cirrhosis complication, bacterial translocation, decompensated cirrhosis, proinflammatory response

## Abstract

Whether the interaction between the gut microbiota and the immune response influences the evolution of cirrhosis is poorly understood. We aimed to investigate modifications of the microbiome and the immune response during the progression of cirrhosis. Rats were treated with carbon tetrachloride (CCl_4_) to induce cirrhosis. We then assessed microbiome load and composition in stool, ileocecal contents (ICCs), mesenteric lymph nodes (MLNs), blood, and ascitic fluids (AFs) at 6, 8, and 10 weeks or ascites production and measured cytokine production in MLNs and blood. The microbiome of MLN, blood, and AF showed a distinct composition compared to that of stool and ICCs. *Betaproteobacteria* (*Sutterella*) were found associated with the appearance of a decompensated state of cirrhosis. Microbial load increased and showed a positive correlation with the relative abundance of pathobionts in the MLN of decompensated rats. Among several genera, *Escherichia* and “*Candidatus* Arthromitus” positively correlated with elevated levels of systemic proinflammatory cytokines. “*Candidatus* Arthromitus,” a segmented filamentous bacteria, was detected in ICC, MLN, and AF samples, suggesting a possible translocation from the gut to the AF through the lymphatic system, whereas *Escherichia* was detected in ICC, MLN, AF, and blood, suggesting a possible translocation from the gut to the AF through the bloodstream. In the present study, we demonstrate that microbiome changes in distinct intestinal sites are associated with microbial shifts in the MLNs as well as an increase in cytokine production, providing further evidence of the role the gut-liver-immunity axis plays in the progression of cirrhosis.

**IMPORTANCE** Cirrhosis severity in patients was previously shown to be associated with progressive changes in the fecal microbiome in a longitudinal setting. Recent evidence shows that bacterial translocation from the gut to the extraintestinal sites could play a major role in poor disease outcome and patient survival. However, the underlying mechanisms involving the microbiota in the disease progression are not well understood. Here, using an animal model of cirrhosis in a longitudinal and multibody sites setting, we showed the presence of a distinct composition of the microbiome in mesenteric lymph nodes, blood, and ascitic fluid compared to that in feces and ileocecal content, suggesting compartmentalization of the gut microbiome. We also demonstrate that microbiome changes in intestinal sites are associated with shifts in specific microbial groups in the mesenteric lymph nodes as well as an increase in systemic cytokine production, linking inflammation to decompensated cirrhosis in the gut-liver-immunity axis.

## Abstract (precis)

Cirrhosis severity in patients was previously shown to be associated with progressive changes in the fecal microbiome in a longitudinal setting. Recent evidence shows that bacterial translocation from the gut to the extraintestinal sites could play a major role in poor disease outcome and patient survival. However, the underlying mechanisms involving the microbiota in the disease progression are not well understood. Here, using an animal model of cirrhosis in a longitudinal and multibody sites setting, we showed the presence of a distinct composition of the microbiome in mesenteric lymph nodes, blood, and ascitic fluid compared to that in feces and ileocecal content, suggesting compartmentalization of the gut microbiome. We also demonstrate that microbiome changes in intestinal sites are associated with shifts in specific microbial groups in the mesenteric lymph nodes as well as an increase in systemic cytokine production, linking inflammation to decompensated cirrhosis in the gut-liver-immunity axis.

## INTRODUCTION

Cirrhosis is defined as the presence of fibrosis and regenerating nodules in the liver due to various causes such as alcohol, hepatitis viruses, metabolic syndrome, or immune dysfunction. Cirrhosis can lead to portal hypertension and liver insufficiency and their related complications, such as ascites, infections, hepatic encephalopathy, hepatorenal syndrome, variceal bleeding, and acute-on-chronic liver failure (ACLF) (1). Before the development of complications, patients are considered to have “compensated cirrhosis,” and when complications develop, they are considered having “decompensated cirrhosis,” which has a poorer prognosis than the compensated stage (2).

Patients with cirrhosis present alterations in their fecal microbiome composition compared to that of healthy individuals, which could be of oral origin of some potentially pathogenic species (3, 4). The cross talk between the gut microbiome and the immune system may contribute to the development of health complications, which therefore may lead to the evolution from compensated to decompensated stages (5, 6).

The intestinal microbiota, which harbors bacteria, archaea, and eukarya, is known to play a pivotal role in the development of the host immune system and in the maintenance of host intestinal homeostasis by modulating immune responses to pathogens and by maintaining the integrity of intestinal barrier functions (7). Commensal bacteria are transported from the intestines by dendritic cells (DCs) through the lymphatic system to the mesenteric lymph nodes (MLNs), which form part of the gut-associated lymphatic tissue (GALT) and act as the first line of immune defense against pathogens from the intestines (8–10). In the MLNs, bacteria are maintained at low levels by the host mucosal immune system (11). The translocation of bacteria into MLNs has been investigated in other disorders, including Crohn’s disease and ulcerative colitis (12, 13), where a distinct microbial community composition was observed between the two inflammatory disorders.

Patients with cirrhosis present alterations in the gut microbiota, intestinal permeability, and immune response, leading to bacterial translocation, which is the paracellular passage of bacteria from the intestinal lumen through the intestinal wall to the MLNs or other sites (10, 11, 14–16). Bacterial translocation then activates the gut-liver-immune axis, which stimulates the induction of proinflammatory cytokines, further perpetuating increased intestinal permeability and thus bacterial translocation (17). Pathogen-associated molecular patterns (PAMPs), such as the endotoxin lipopolysaccharide (LPS) found on the cell membranes of Gram-negative bacteria, bind to pattern recognition receptors (PRRs) such as Toll-like receptors (TLRs), causing an induction of proinflammatory cytokines such as tumor necrosis factor alpha (TNF-α) and interleukin 6 (IL-6), which tend to be elevated in patients with cirrhosis (18). MLNs also produce TNF-α in response to bacterial translocation, especially in patients with ascites (9, 18, 19). Ascites is a common complication in advanced cirrhosis that is associated with a high mortality rate and is caused by portal hypertension, leading to fluid accumulation in the abdomen. It has been hypothesized that elevated TNF-α production causes hemodynamic disturbances, leading to splanchnic vasodilatation through nitric oxide synthesis stimulation. This could contribute to altered intestinal barrier function, resulting in bacterial translocation (9, 20), which has been frequently observed in cirrhotic patients with ascites. Additionally, ascites has been shown to increase the susceptibility of host bacterial infection (17), likely due to the fact that TNF-α has been shown to loosen tight junction proteins of intestinal epithelial cells, perpetuating bacterial translocation and subsequently an inflammatory response (10).

Bajaj et al. showed that cirrhosis severity in patients was associated with progressive changes in the gut microbiome in a longitudinal study (5). In fact, recent evidence showed that bacterial translocation from the intestines could play a major role in poor disease outcome and patient survival (21, 22). However, the underlying mechanisms that involve the gut microbiota in the disease progression are not well understood.

Therefore, the aims of this study were to (i) investigate the spatial and temporal changes of the composition of the microbiome in a cirrhosis rat model, (ii) evaluate changes of the microbiome related to the progression of cirrhosis, and (iii) assess the immune modulation by the microbiome detected in extraintestinal sites.

## RESULTS

We studied 15 control rats at weeks 6 (*n *=* *5), 8 (*n *=* *5), and 10 (*n *=* *5), 25 CCl_4_-treated rats sacrificed at weeks 6 (*n *=* *9), 8 (*n *=* *8), and 10 (*n *=* *8) of CCl_4_ cirrhosis induction that were considered rats under induction of cirrhosis or compensated CCl_4_-treated rats, and 19 CCl_4_-treated rats when they developed ascites that were considered rats with decompensated cirrhosis. The scores of liver damage in the different groups were the following. All control rats showed score 0; in week-6 rats, six rats had scores of 2 and three had scores of 1. In week-8 rats, six rats had scores of 2 and two had scores of 1. In week-10 rats, seven rats had scores of 2 and one had a score of 1. In ascitic rats, 14 rats had scores of 2 and five had scores of 3 (*P *= 0.048 for week-10 rats, *P *= 0.019 for week-8 rats, and *P *= 0.007 for week-6 rats). Bacterial cultures were negative in all control rats, week-6 rats, and week-8 rats and were positive in two week-10 rats (both in MLNs) and in five ascitic rats (*P *= 0.05 between ascitic rats and control rats). These five ascitic rats presented a total of nine positive cultures: in MLNs in four rats, in livers and spleens in three rats, and in ascitic fluid in two rats. Isolated bacteria were Escherichia coli in all rats with positive cultures, except one ascitic rat in which *Enterococcus* spp. grew.

### Compartmentalization of microbial communities.

**FIG 1:**
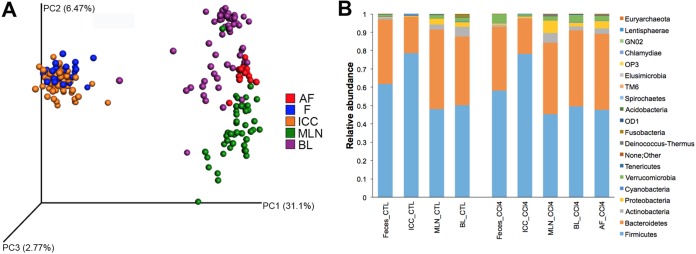
Spatial microbial community compositions in control and CCl_4_-treated rats. (A) Weighted principal coordinate analysis (PCoA) UniFrac metrics (taxonomic clustering). (B) Relative abundance at the phylum level; *n *=* *23 for stool, *n *=* *59 for ICC, *n *=* *46 for MLN, *n *=* *57 for blood, and *n *=* *15 for AF.

To assess the existence of a compartmentalized microbiome (meaning a specific microbial community at different body sites), we compared the microbiome compositions of various body sites from all rats of the study: intestinal sites such as feces (F) and ileocecal content (ICC) and extraintestinal sites such as MLN, blood, and ascitic fluid (AF). Feces and ICC specimens from control rats presented similar microbial communities (*P = *0.44; permutational multivariate analysis of variance [PERMANOVA] test) (Fig. 1AB) dominated by *Firmicutes* (means of 61% and 78%, respectively), *Bacteroidetes* (means of 35% and 20%, respectively), *Actinobacteria* (means of 1% and 0.04%, respectively), and *Proteobacteria* (means of 0.3% and 0.4%, respectively). The microbiome of MLN, blood, and AF showed a distinctive composition compared to that of feces and ICC (*P < *0.0001; PERMANOVA test) (Fig. 1A). MLNs and blood displayed similar microbial communities (*P = *0.616; PERMANOVA test) dominated by *Firmicutes* (48% and 50%, respectively), *Bacteroidetes* (43% and 37%, respectively), *Proteobacteria* (3% and 2%, respectively), and *Actinobacteria* (3% and 5%, respectively) (Fig. 1B). A comparison between control and CCl_4_-treated rats in the different body sites showed significant differences.

### Longitudinal study: evolution of cirrhosis and microbiome modification.

To evaluate the evolution of the microbiome in parallel with the progression of cirrhosis, rats were sacrificed to collect ICC, MLNs, and blood at different time points (6, 8, and 10 weeks after the initiation of CCl_4_ treatment and at ascites development for the CCl_4_-treated group and at matched time points for the control group). For the collection of samples, groups of animals were sacrificed at different time points. We found significant changes in the microbiome compositions in ICC and MLN samples that were associated with the progression of cirrhosis.

**FIG 2:**
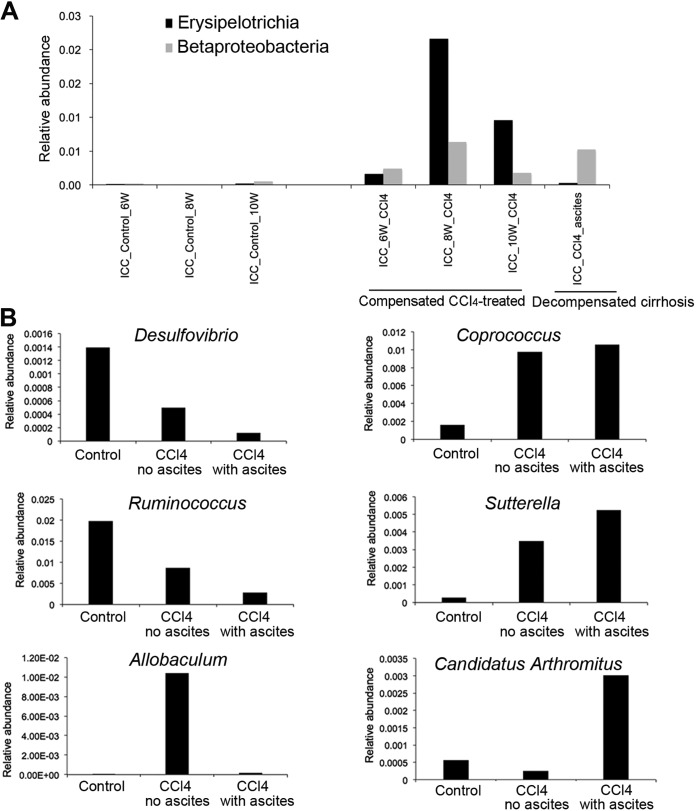
Microbial groups in ileocecal contents (ICC) samples involved in the severity of cirrhosis. (A) Temporal taxonomic differences between controls and CCl_4_-treated rats. Two classes of bacteria, *Erysipelotrichia* (*P *= 0.001, *q *=* *0.012) and *Betaproteobacteria* (*P *= 0.01, *q *=* *0.098), presented significantly different relative abundances over time between the control and the CCl_4_-treated groups. Statistics were performed using the Kruskal-Wallis test. (B) Taxonomic differences between controls and CCl_4_-treated rats with ascites and CCl_4_-treated rats before development of ascites. Two bacterial genera, *Coprococcus* (*P *= 0.0001, *q *=* *0.011) and *Sutterella* (*P *= 0.0005, *q *=* *0.014), were found in higher relative abundances in CCl_4_-treated rats than in control rats. Two bacterial genera, *Desulfovibrio* (*P *= 0.002, *q *=* *0.025) and *Ruminococcus* (*P *= 0.0007, *q *=* *0.013), were found in higher relative abundances in control rats than in CCl_4_-treated rats. *Allobaculum* was found in higher relative abundance (*P *= 0.0004, *q *=* *0.013) only in compensated CCl_4_-treated rats, and “*Candidatus* Arthromitus” (*P *= 0.023, *q *=* *0.15) was in higher relative abundance in decompensated cirrhotic rats. Statistics were performed using the Kruskal-Wallis test.

In the ICC samples, CCl_4_-treated rats showed an increase in *Betaproteobacteria* (*P = *0.01, *q *=* *0.098) and *Erysipelotrichia* (*P = *0.001, *q *=* *0.012; Kruskal-Wallis test) at weeks 6 and 8 of CCl_4_ treatment in comparison to control rats (Fig. 2A). At week 10, both groups of bacteria significantly decreased compared to that at weeks 6 and 8. When rats developed ascites, *Erysipelotrichia* almost disappeared, whereas *Betaproteobacteria* increased again. Both groups of bacteria were absent in control rats. At the genus level, *Sutterella* and *Coprococcus* increased (*P < *0.001, *q *<* *0.02; Kruskal-Wallis test) in decompensated rats compared to that in controls (Fig. 2B). At 6, 8, and 10 weeks after the initiation of CCl_4_ treatment, rats without ascites presented an intermediate relative abundance of all these genera. The *Allobaculum* genus belonging to the *Erysipelotrichia* family is known as a potentially beneficial bacterial group (23) and showed an increase in the compensated CCl_4_-treated rats but disappeared when they presented ascites (*P = *0.0002, *q *=* *0.018; Kruskal-Wallis test). These results suggest that groups of potentially beneficial bacteria, such as *Allobaculum*, attempted to outcompete pathogenic ones in the ileocecum such as *Sutterella*, which belongs to *Betaproteobacteria*. However, *Allobaculum* seemed to be unable to outcompete *Sutterella* in the ICC when rats presented ascites. Additionally, “*Candidatus* Arthromitus,” a genus from the *Firmicutes* phylum, showed high relative abundance only in decompensated rats compared to that in compensated CCl_4_-treated rats and control rats (*P = *0.023, *q *=* *0.15; Kruskal-Wallis test) (Fig. 2C).

**FIG 3:**
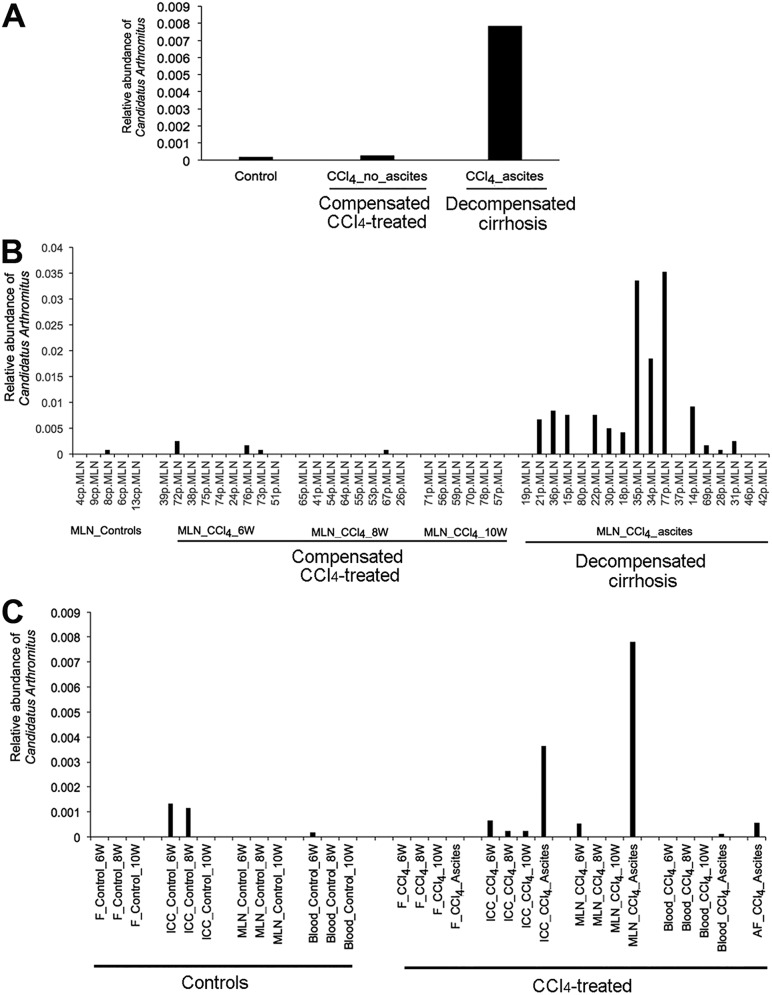
Detection of “*Candidatus* Arthromitus” in spatial and temporal settings. (A) “*Candidatus* Arthromitus” was found in higher relative abundance in mesenteric lymph nodes of decompensated rats (*P *= 0.0002, *q* = 0.018). (B) “*Candidatus* Arthromitus” was found in 72% (13 of 18) of mesenteric lymph node samples of decompensated rats. (C) “*Candidatus* Arthromitus” was detected in ileocecal content (ICC), mesenteric lymph node (MLN), blood, and ascitic fluid (AF) samples but not in feces (F). 6W, 6 weeks; 8W, 8 weeks; 10W, 10 weeks.

In MLN samples from decompensated rats (with ascites), only one bacterial genus, “*Candidatus* Arthromitus,” showed a significantly high relative abundance compared to that from control and compensated CCl_4_-treated rats (*P = *0.0002, *q *=* *0.019; Kruskal-Wallis test) (Fig. 3A and B). “*Candidatus* Arthromitus” was identified in 26% (4 of 15) of the AF samples, in only 5% of blood samples (4 of 73), and was not found in stool samples (Fig. 3C).

In feces, *Coprococcus, Sutterella*, and *Allobaculum* showed some differences in their relative abundances between the three groups of rats (controls, compensated, and decompensated), but none of the differences were significant (*q *>* *0.2; Kruskal-Wallis test). In blood samples, the *Spirochaetes* phylum was found in a high proportion in the decompensated group compared to that in the two other groups (*P = *0.001, *q *=* *0.035; Kruskal-Wallis test).

Using the weighted UniFrac distance, a metric used to compare microbial community compositions between samples, we compared the stability of the microbiome in ICC, MLN, and blood samples from rats under induction of cirrhosis without ascites (compensated CCl_4_-treated rats) with that from rats with ascites (rats with decompensated cirrhosis). We observed a decreased stability of the microbiome composition of rats with ascites in blood samples (*P* = 0.04; Mann-Whitney test) (see Fig. S1 in the supplemental material), but the difference was not significant in ICC and MLN samples.

10.1128/mSystems.00278-18.1 FIG S1 Microbiome instability. Weighted UniFrac distances were calculated between different time periods (weeks 6, 8, and 10) for cirrhotic rats producing ascites and CCl_4_-treated rats without ascites. UniFrac indexes obtained at week 6 were compared to those at weeks 8 and 10 or at time of ascites production. Progression of cirrhosis to ascites was not associated with instability of the microbiome composition in ICC nor in MLN (*P > *0.34; Mann-Whitney test) but was associated in blood samples (*P *= 0.04; Mann-Whitney test). A larger UniFrac index means greater distance and therefore greater instability. Download FIG S1, TIF file, 0.4 MB. Copyright © 2019 Santiago et al. 2019 Santiago et al. This content is distributed under the terms of the Creative Commons Attribution 4.0 International license.

### Microbial load.

**FIG 4:**
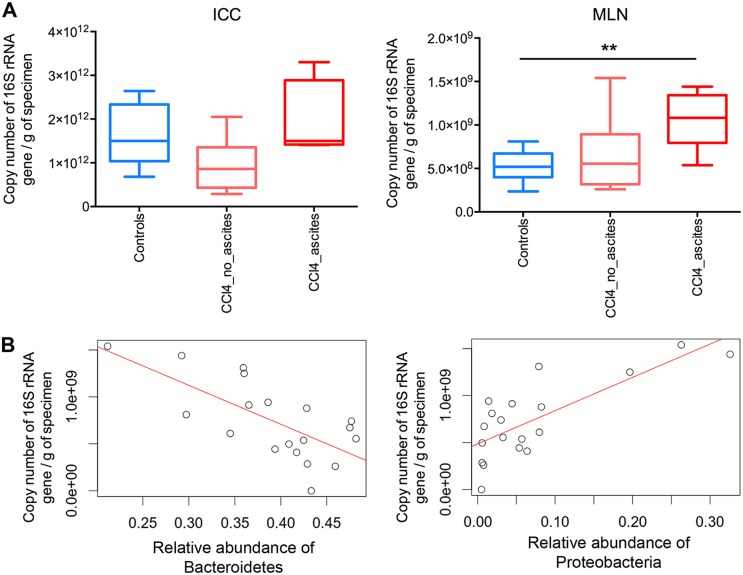
Quantification of the microbiota by real-time PCR on the 16S rRNA gene and correlation with microbiome composition. (A) Microbial load in ileocecal content (ICC) samples was higher in decompensated cirrhotic rats than in control and compensated CCl_4_-treated rats and was significantly higher in the mesenteric lymph nodes (MLNs) of decompensated cirrhotic rats than in control rats (*P *= 0.008; Mann-Whitney test). In both ICCs and MLNs: *n *=* *8 for controls (CTL), *n *=* *8 for compensated CCl_4_-treated rats, *n *=* *7 for decompensated cirrhotic rats. (B) Spearman correlation between microbial load and relative abundance of *Bacteroidetes* (ρ = −0.637, *P *= 0.004) and between microbial load and relative abundance of *Proteobacteria* (ρ = 0.673, *P *= 0.002) in the MLNs.

To complement our findings on relative abundance of the sequenced 16S rRNA genes, we evaluated the microbial load using real-time quantitative PCR of the 16S rRNA genes. Microbial load, as measured by 16S rRNA gene quantitative PCR (qPCR), was significantly higher in the MLNs from cirrhotic decompensated rats than from control rats (*P = *0.008; Mann-Whitney test), positively correlated with the relative sequence abundance of *Proteobacteria* (ρ = 0.673, *P = *0.002; Spearman test), and negatively correlated with the relative abundance of *Bacteroidetes* (ρ = −0.637, *P = *0.004; Spearman test) (Fig. 4).

### Correlation between microbiome and cytokine levels.

**FIG 5:**
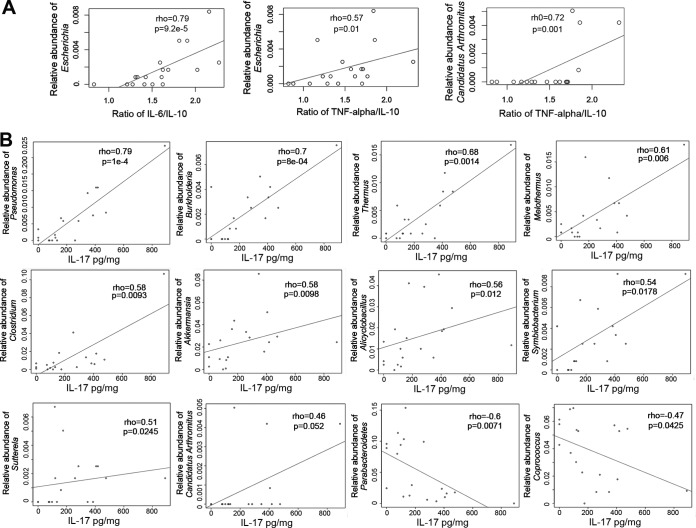
Correlations between proinflammatory cytokines and relative abundances of genera in MLNs. (A) Positive Spearman correlations were found between the ratio of systemic IL-6/IL-10 and *Escherichia* and between the ratio of systemic TNF-α/IL-10 and *Escherichia* and “*Candidatus* Arthromitus.” (B) Spearman correlations between IL-17 levels measured in mesenteric lymph nodes (MLNs) and relative abundances of genera detected in MLNs.

**FIG 6:**
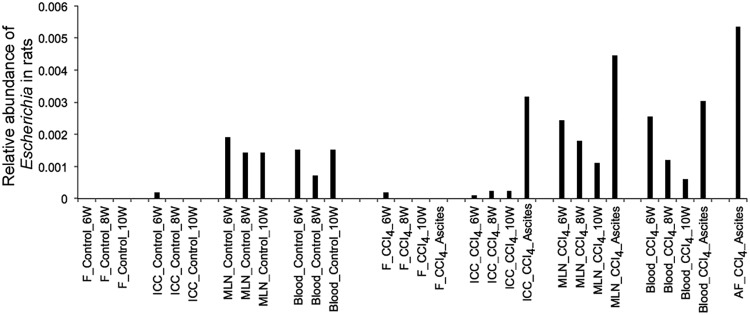
Detection of 16S sequences of *Escherichia* in rat samples. ICC, ileocecal content; MLN, mesenteric lymph node; AF, ascitic fluid; F, feces.

The progression toward decompensated cirrhosis is associated with a high production of proinflammatory cytokines, such as TNF-α, IL-17, and IL-6, as well as anti-inflammatory cytokines such as IL-10 (24, 25). Using enzyme-linked immunosorbent assays (ELISAs), we measured serum and MLN levels of these cytokines. To evaluate a possible correlation between the inflammatory status and the microbial community composition, we used Spearman correlation tests to associate levels of the proinflammatory cytokine IL-17 in MLNs and the ratios of systemic IL-6/IL-10 and systemic TNF-α/IL-10 with the relative abundance of microbial groups in MLNs. Ratios between pro- and anti-inflammatory cytokines have been extensively used as biomarkers to associate an immune response with the characteristics of multiple pathologies, including liver disease. The use of ratios reduces significantly the individual variability of single cytokine production. We found a positive correlation between the IL-6/IL-10 ratio and the relative abundance of *Escherichia* (ρ = 0.79, *P = *9.2e−5; Spearman test) and positive correlations between the TNF-α/IL-10 ratio and the relative abundances of *Escherichia* (ρ = 0.57, *P = *0.01; Spearman test) and “*Candidatus* Arthromitus” (ρ = 0.72, *P = *0.001; Spearman test) (Fig. 5A). Since each microorganism provides a particular array of PAMPs to signal the immune system, the final result is not necessarily a generalized increase of pro- or anti-inflammatory cytokines. In the particular case of “*Candidatus* Arthromitus,” the association of one ratio but not the other (both having in common IL-10) implies that this microorganism is more involved in the production/regulation of TNF than of IL-6. We also found positive correlations between levels of IL-17 and several different microbial genera, such as those belonging to *Proteobacteria* (*Pseudomonas*, *Burkholderia* and *Sutterella*) and “*Candidatus* Arthromitus,” and negative correlations between levels of IL-17 and *Parabacteroides* and *Coprococcus* (Fig. 5B). “*Candidatus* Arthromitus” and *Escherichia* were both detected in ICC, MLN, and AF samples, but only *Escherichia* was detected in blood samples from rats (Fig. 6).

## DISCUSSION

In this study, we showed that microbiome changes in distinct intestinal sites are associated with microbial shifts in the MLNs as well as an increase in cytokine production that correlated with disease progression in rats with experimental cirrhosis. The main results of the present work are the characterization of the sequential changes of the microbiome in the progression of cirrhosis and, in particular, the cross talk between the microbiome and the host immune system using a rat model in a longitudinal and multibody sites study setting.

Loss of intestinal barrier function, dysbiosis, and systemic immune dysfunction characterize cirrhosis (17). Bacterial translocation is a result of a loss of intestinal barrier function and is considered a biomarker for cirrhosis progression and decompensation, in which intestinal bacteria travel by paracellular transport through the permeable epithelial cells to the portal vein, the liver, and systemic circulation, causing an inflammatory response (10). It has been demonstrated that microorganisms are also transported by dendritic cells from the intestines to the MLNs via the lymphatic system (11, 26, 27). Comparing the compositions of intestinal and extraintestinal body sites, our findings suggest a loss of barrier function in which a specific microbial community, particularly *Proteobacteria* and *Actinobacteria*, was transported from the intestine to MLNs, blood, and AF, which caused an induction of proinflammatory cytokines such as TNF-α, IL-6, and IL-17 in rats. This inflammatory response was associated with disease progression and decompensation with ascites formation.

It has been suggested that dysbiosis, which is the unfavorable shift in the microbiota community structure that compromises microbe-host homeostasis, is a major driver of cirrhosis and is also a major contributor to bacterial translocation to MLNs in cirrhosis animal models (10, 28). In the present study, we found more microbiome alterations in ICC than in fecal samples of rats with decompensated cirrhosis. This suggests that ICC samples may be more appropriate than fecal samples to detect microbiome composition alterations with smaller cohorts. This is in line with previous studies in patients suggesting that dysbiosis in advanced cirrhosis mainly occurs in upper areas of the intestine such as the ileum (10) as well as with the finding that microbiota analyses in fecal samples do not accurately represent dysbiosis in sigmoid mucosal samples obtained by biopsy during sigmoidoscopy (29). In the present study, ICC samples from CCl_4_-treated rats showed an attempt of beneficial groups of bacteria, such as *Erysipelotrichia*, to outcompete potential pathobionts such as *Sutterella*, which belongs to *Betaproteobacteria*. A pathobiont is defined as a symbiont that under certain circumstances becomes a pathogen (30). This compensation failed when liver damage and inflammation increased and rats developed ascites, a condition associated with an increase of inflammatory markers, an increase in the relative abundance of *Escherichia* in all sample types except in stool, and an increase in the load of *Proteobacteria* in MLNs.

Bajaj et al. (5) characterized the composition of the fecal microbiome in patients with compensated and decompensated cirrhosis and also evaluated the stability of the microbiome composition at two time points within an interval of 6 months. Our findings confirmed their claim that the level of dysbiosis in stool samples was associated with cirrhosis progression, whereas a relatively stable microbiome composition over time was associated with stable disease. Additionally, the use of an animal model allowed us to unravel the possible cross talk between the microbiome in MLNs and the immune system that was confirmed through a correlation analysis between microbial genera identified in MLNs and levels of proinflammatory cytokines such as TNF-α, IL-6, and IL-17 in MLNs or blood. Moreover, our results are congruent with a comprehensive study that analyzed 29 different cytokines from 522 cirrhotic patients, indicating that systemic inflammation is likely the underlying cause of decompensation and acute-on-chronic liver failure in cirrhosis (8). Finally, our findings are supported by the recent work of Muñoz et al. (6) that also demonstrated the appearance of a proinflammatory immune response driven by gut dysbiosis in decompensated cirrhosis.

We also observed a positive correlation between the abundance of *Escherichia*, which belongs to the *Proteobacteria* phylum, in MLNs and proinflammatory cytokines, confirming the results of previous studies using culture techniques and PCR of the 16S rRNA gene and making *Escherichia* a potential biomarker for cirrhosis progression (31). We have further demonstrated that an increase in overall microbial load was associated with an increase in *Proteobacteria* in the MLNs of decompensated cirrhotic rats compared to that in controls and in compensated CCl_4_-treated rats, suggesting an increase in *Proteobacteria* not only in relative abundance but also in absolute amount.

Furthermore, we observed that “*Candidatus* Arthromitus,” a genus from the *Firmicutes* phylum that positively correlated with IL-17 levels, was found in higher abundance in the MLNs of rats with decompensated cirrhosis than in control rats and compensated CCl_4_-treated rats. This genus was detected in all sample types except feces, which again confirms that the use of stool samples would not have led to the detection of this genus in a decompensated state. “*Candidatus* Arthromitus” is a segmented filamentous bacterium (SFB) that can induce multiple adaptive immune responses, especially in Th17 cells in the small intestinal lamina propria of mice (32, 33). Th17 cells that produce the effector cytokine IL-17 are potent inducers of tissue inflammation and have been associated with the pathogenesis of many immune-mediated diseases (34). Therefore, “*Candidatus* Arthromitus” might play an important role in inducing inflammation, which may lead to a decompensated state. This genus is also well known to be refractory to *in vitro* culture techniques (35, 36), which explains why it has not been uncovered in previous studies using traditional culture techniques. This genus, which was not detected in feces and in only few blood samples, might have reached the MLNs via the lymphatic system after possible translocation in the upper regions of the gastrointestinal (GI) tract, such as the ileocecal region. *Escherichia*, differently from “*Candidatus* Arthromitus,” was detected in all sample types, including in all blood samples, suggesting that its translocation to AF might be via the bloodstream.

Since this work was performed on animals, we may not be able to extrapolate all the findings to cirrhotic patients, particularly in terms of the involvement of specific genera such as “*Candidatus* Arthromitus,” as the microbiome might present differences in its composition. However, our study should pave the way for the search for an equivalent genus to “*Candidatus* Arthromitus” in humans that is involved in inducing inflammation in cirrhotic patients. Also, the limited numbers of animals per group might be the reason why we did not find significant changes in fecal samples after multiple testing correction but rather a trend, compared to that of previous findings in human fecal samples (4, 37). Another limitation of our study might be the use of a unique liver disease animal model, as different etiologies might lead to different changes in the microbiome as shown by Fouts et al. (38). However, although different microbial groups might be involved in the progression of cirrhosis depending on the animal model used, our study mainly focused on the sequential changes in the microbiome in distinct intestinal sites and their association with inflammation in an advanced stage of decompensated cirrhosis. This limitation may apply to any animal models used to understand human disease.

In conclusion, our study confirmed previous studies showing that the alterations in the gut microbial community involved an increase of the ratio of pathobionts to beneficial bacteria (39) and also showed that this reflects dysbiosis present in extraintestinal sites such as MLNs, where direct cross talk between the microbiota and the immune cells takes place.

## MATERIALS AND METHODS

### Experimental design.

**(i) Animals.** Male Sprague-Dawley rats weighing 35 to 49 g were purchased from Harlan Laboratories (Indianapolis, IN, USA) and provided by Research Models and Services Production (Udine, Italy). After the rats were weaned from their mothers, they were fed a rodent chow diet (2018S; Teklad, Madison, WI, USA). After 1 week of quarantine, all animals were placed in individual cages and kept at a constant room temperature of 21°C, exposed to a 12-h light:12-h dark cycle and allowed free access to water and rodent chow (A04; SAFE, Augy, France). One week later, phenobarbital (1.5 mmol/liter) (Luminal; Kern Pharma, Barcelona, Spain) was added to the tap water given to all animals. There was no contact between rats via water, chow, or feces.

**(ii) Induction of cirrhosis and study groups.** Cirrhosis was induced as previously described (40). When rats reached a weight of 200 g, they were administered weekly doses of CCl_4_ (Sigma-Aldrich, St. Louis, MO., USA) intragastrically using a sterile pyrogen-free syringe (ICO plus 3; Novico Médica, S.A., Barcelona, Spain) with an attached stainless-steel animal feeding tube (Popper and Sons, New Hyde Park, NY, USA) without anesthesia. The first dose of CCl_4_ was 20 μl, and subsequent doses were adjusted on the basis of changes in weight 48 h after the previous dose. When rats presented ascites, the dose of CCl_4_ was maintained at 40 μl.

We designed different groups of CCl_4_-treated rats for which laparotomy and sample collection were performed at four different time points: after 6, 8, and 10 weeks of the first dose of CCl_4_ and when ascites was suspected by the increase in abdominal girth and confirmed by paracentesis. A control group of rats not treated with CCl_4_ was also included, and samples were collected at the same three time points (6, 8, and 10 weeks) as for the cirrhotic group.

Paracentesis was performed under air anesthesia with isoflurane (Forane; Abbott, Madrid, Spain) under sterile conditions, and approximately 0.1 ml of ascitic fluid was removed. One week later, a laparotomy was carried out.

**(iii) Laparotomy.** Laparotomy was carried out on all CCl_4_-treated rats and on control rats at weeks 6, 8, and 10 or when ascites was suspected. For laparotomy, rats were anesthetized with 10 mg/kg xylazine (Rompun; Bayer, Kiel, Germany) and 50 mg/kg ketamine (Ketolar; Parke-Davis, Madrid, Spain) under sterile conditions. In brief, the abdominal fur was removed with a depilatory cream (Deliplus; Mercadona, Spain) and the skin was sterilized with iodine (Curadona; Lainco, Spain). The abdomen was then opened via a 4-cm median incision, and the remaining fluid was removed.

### Biological sample collection.

The sequence of sample collection at laparotomy was stool (before laparotomy), MLN, blood, liver spleen, and ileocecal content (ICC). Samples from CCl_4_-treated and control rats were stored frozen at −80°C until microbiome analysis. Blood and MLN samples were also used for cytokine analysis.

### Bacterial cultures.

We inoculated samples of homogenized mesenteric lymph nodes, ascitic fluid, pleural fluid, spleens, and livers on Columbia blood agar, Columbia CNA agar, and the chromogenic medium CPS ID3 (bioMérieux, Marcy-l’Étoile, France). Cultures were incubated for 48 h at 37°C in an aerobic atmosphere. The isolated bacteria were presumptively identified according to their pattern of growth and morphology (41).

### Liver damage.

Histological liver damage was evaluated by hematoxylin-eosin and Masson’s trichrome staining of 4-µm slices from paraffin blocks. A single expert pathologist blindly classified the liver samples according to a semiquantitative score: 0, normal; 1, fibrosis with porto-portal fibrous tracts; 2, regeneration nodules with thin complete fibrous tracts; and 3, regeneration nodules with thick and complete fibrous tracts (41).

### Cytokine measurement.

TNF-α, IL-6, and IL-10 cytokines were determined in blood samples and IL-17 in MLNs by enzyme-linked immunosorbent assays (ELISAs) according to the manufacturer’s protocols (eBiosciences). Results are expressed as picograms per milliliter in blood samples and the ratio of picograms of IL-17 per milligram of total protein. Limits of detection were 30 pg/ml for TNF-α, IL-6, and IL-17 and 15 pg/ml for IL-10.

### Microbiome analysis.

**(i) Genomic DNA extraction.** All biological specimens were processed for genomic DNA extraction using protocols previously described by Santiago et al. (42) for low biomass samples such as MLN, blood, and AF and a protocol recommended by the International Human Microbiome Standard for stool samples (http://www.microbiome-standards.org/).

**(ii) 16S rRNA genes gene sequencing.** To prepare the DNA for sequencing, we amplified a fragment of the 16S rRNA gene by PCR using universal primers targeting the V4 hypervariable region as previously described (43). Amplicons were then purified using the QIAquick PCR purification kit (Qiagen, Barcelona, Spain), quantified using a NanoDrop ND-1000 spectrophotometer (Nucliber), and then pooled in equal concentrations. The pooled amplicons (2 nM) were then subjected to sequencing using Illumina MiSeq technology at the technical support unit of the Autonomous University of Barcelona (UAB, Spain) according to standard Illumina platform protocols.

**(iii) Microbiome composition analysis.** To analyze the microbiome composition, we first loaded the raw sequences into the QIIME 1.9.1 pipeline, as described by Navas-Molina et al. (44). Low-quality sequence reads were filtered out by applying default settings and a minimum Phred score of 20. From the filtering step and a total of 214 samples, we obtained a total of 2.5 million high-quality sequences with an average number of reads of 11,899. We used the USEARCH algorithm to cluster similar filtered sequences into operational taxonomic units (OTUs) based on a 97% similarity threshold. We then identified and removed chimeric sequences using UCHIME. Representative sequences were selected and aligned using PyNAST against Greengenes template alignment (gg_13_8 release), and a taxonomical assignment step was performed using the basic local alignment search tool to map each representative sequence against a combined database encompassing the Greengenes and PATRIC databases.

For β diversity analysis, we rarefied to 1,046 sequences per sample when comparing all samples simultaneously. When analyzing only low-biomass samples, we rarefied them at 1,046 sequences per sample and at 9,396 sequences per sample when analyzing stool and ICC samples. Rarefaction is used for cases in which read counts are not similar in numbers between samples. Weighted and unweighted UniFrac metrics were applied to build phylogenetic distance matrices, which were then used to construct hierarchical cluster trees using a principal-coordinate analysis (PCoA) representation.

**(iv) Microbial load assessment.** To quantify microorganisms, the extracted genomic DNA was used to amplify the V4 region of the 16S rRNA gene by quantitative real-time PCR (qPCR) using the following primers: V4F_517_17 (5′-GCCAGCAGCCGCGGTAA-3′) and V4R_805_19 (5′-GACTACCAGGGTATCTAAT-3′). To calibrate the qPCR reactions, calculated amounts of a linearized plasmid containing the V4 region of the 16S rRNA gene were used. Plasmid concentration was measured using a NanoDrop ND-1000 spectrophotometer (Nucliber), and the number of plasmid copies was calculated from the plasmid’s molecular weight. To extrapolate the bacterial number in each sample, serial dilutions of the plasmid were amplified. The qPCR was performed with the 7500 Fast Real-Time PCR system (Applied Biosystems) using optical-grade 96-well plates. The PCR was performed in a total volume of 25 µl using the Power SYBR green PCR master mix (Applied Biosystems) containing 100 nM each of the universal forward and reverse primers. The reaction conditions for amplification of DNA were 50°C for 2 min, 95°C for 10 min, and 40 cycles of 95°C for 15 s and 60°C for 1 min. All reactions were performed in triplicates, and mean values were calculated. This experiment was also duplicated to ensure accuracy. Mean values from both experiments were taken into account. Data were analyzed using Sequence Detection Software version 1.4, supplied by Applied Biosystems.

### Statistical analysis.

We performed statistical analyses under QIIME and R. We used the D’Agostino-Pearson test to check for the normality of the data distribution. Parametric normally distributed data were compared by Student's *t* tests for paired or unpaired data; otherwise, the Wilcoxon signed rank test was used for paired data and the Mann-Whitney U test for unpaired data. Qualitative variables were analyzed by Fisher’s exact test. The Kruskal-Wallis one-way test of variance was used to compare the mean numbers of sequences from different unpaired groups of subjects at various taxonomic levels, the Wilcoxon test was used when comparing only 2 groups. We performed analyses with the nonparametric multivariate ANOVA (PERMANOVA) called the Adonis test to test for differences in microbial communities using the UniFrac metrics. We performed Spearman tests to evaluate correlations between microbiome composition and biological parameters such as cytokine levels. When possible, the analysis provided false discovery rate (*q*)-corrected *P* values (*q* values). A *q* value of <0.05 was considered significant for all tests.

### Ethics approval.

The study was approved by the Animal Research Committee at the Institut de Recerca of Hospital de la Santa Creu i Sant Pau (Barcelona) and by the Department of Agriculture, Livestock and Fisheries of the Generalitat de Catalunya (Departament d'Agricultura, Ramaderia i Pesca). Animal care complied with the criteria outlined in the Guide for the Care and Use of Laboratory Animals (Committee for the Update of the Guide for the Care and Use of Laboratory Animals Institute for Laboratory Animal Research Division on Earth and Life Studies, Washington, DC, USA).

### Data availability.

Sequence data have been deposited in the NCBI database under accession number PRJNA448565.
