# Supplementary figures and images for: Sequential Changes in the Mesenteric Lymph Node Microbiome and Immune Response during Cirrhosis Induction in Rats
Source: mSystems. 2019 Feb 19;4(1):e00278-18. doi: 10.1128/mSystems.00278-18 (PMC6381228; doi:10.1128/mSystems.00278-18)

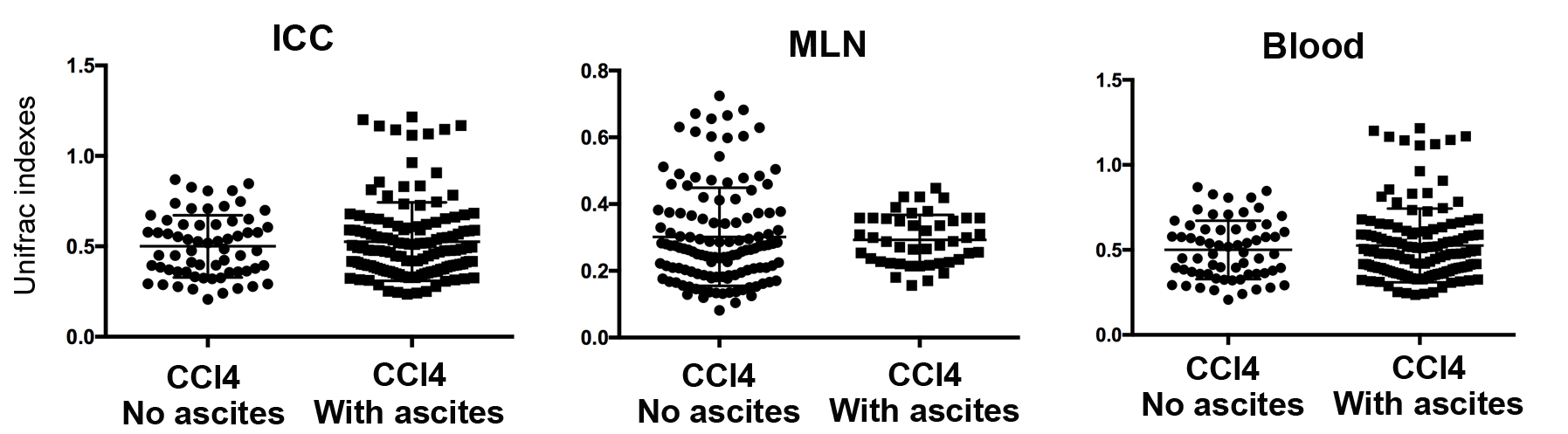

Supplement: FIG S1 [file mSystems.00278-18-sf001.tif]
